# Non-coding RNA in the gut of the blood-feeding parasitic worm, *Haemonchus contortus*

**DOI:** 10.1186/s13567-023-01254-x

**Published:** 2024-01-03

**Authors:** Caixian Zhou, Waresi Tuersong, Lu Liu, Wenda Di, Li He, Fangfang Li, Chunqun Wang, Min Hu

**Affiliations:** 1 State Key Laboratory of Agricultural Microbiology, College of Veterinary Medicine, Huazhong Agricultural University, Wuhan, 430070 Hubei China; 2 College of Veterinary Medicine, Xinjiang Agricultural University, Wulumuqi, 830052 Xinjiang China; 3 College of Animal Science and Technology, Guangxi University, Nanning, 530004 Guangxi China; 4 School of Basic Medical Sciences, Hubei University of Medicine, Hubei, 442000 Shiyan China; 5 College of Biology and Food Engineering, Chongqing Three Gorges University, Chongqing, 402020 China

**Keywords:** *Haemonchus contortus*, intestine, long non-coding RNA, circRNA, miRNA, ceRNA

## Abstract

The intestine of *Haemonchus contortus* is an essential tissue that has been indicated to be a major target for the prevention of haemonchosis caused by this parasitic nematode of small ruminants. Biological peculiarities of the intestine warrant in-depth exploitation, which can be leveraged for future disease control efforts. Here, we determined the intestinal ncRNA (lncRNA, circRNA and miRNA) atlas using whole-transcriptome sequencing and bioinformatics approaches. In total, 4846 novel lncRNA, 982 circRNA, 96 miRNA (65 known and 31 novel) and 8821 mRNA were identified from the *H. contortus* intestine. The features of lncRNA, circRNA and miRNA were fully characterized. Comparison of miRNA from the intestines and extracellular vesicles supported the speculation that the miRNA from the latter were of intestinal origin in *H. contortus*. Further function analysis suggests that the *cis*-lncRNA targeted genes were involved in protein binding, intracellular anatomical structure, organelle and cellular process, whereas the circRNA parental genes were mainly enriched in molecular function categories, such as ribonucleotide binding, nucleotide binding, ATP binding and carbohydrate derivative binding. The miRNA target genes were related to the cellular process, cellular response to stimulus, cellular protein modification process and signal transduction. Moreover, competing endogenous RNA network analysis revealed that the majority of lncRNA, circRNA and mRNA only have one or two binding sites with specific miRNA. Lastly, randomly selected circRNA, lncRNA and miRNA were verified successfully using RT-PCR. Collectively, these data provide the most comprehensive compilation of intestinal transcripts and their functions, and it will be helpful to decipher the biological and molecular complexity of the intestine and lay the foundation for further functional research.

**Supplementary Information:**

The online version contains supplementary material available at 10.1186/s13567-023-01254-x.

## Introduction

One of the most compelling discoveries of the genome era of research is that the genome is pervasively transcribed from noncoding regions [1]. Based on the length, they are mainly categorized into two classes: short non-coding RNA (< 200 nt), such as ribosomal RNA (rRNA), transfer RNA (tRNA), microRNA (miRNA), small interfering RNA (siRNA), piwi-interacting RNA (piRNA), and long non-coding RNA (> 200 nt) containing lncRNA and circular RNA (circRNA) [2]. Among these ncRNA, miRNA are single-stranded yet powerful regulatory RNA molecules ranging in length from 20–24 nt, which can promote mRNA degradation or translation inhibition based on complete or incomplete pairing with mRNA 3ʹ-UTR via RNA-induced silencing complexes [2]. Compared with miRNA, lncRNA have been discovered to be involved in regulating a series of biological processes at transcriptional, post-transcriptional and epigenetic levels through mechanisms interacting with DNA, RNA or proteins [2]. In contrast, circRNA are a group of endogenous validated regulatory ncRNA species with covalently closed circular structures and more stable than linear RNA, which can function as miRNA or RNA-binding protein sponges, and regulators of splicing and transcription [3].

An accumulating list of ncRNA has been reported to be critical regulators in various biological processes in many species, including helminths. For miRNA, Csi-let-7a-5p facilitates the activation of M1-like macrophages and the biliary injuries by targeting Socs1 and Clec7a in *Clonorchis sinensis* [4]. The miR-228 and miR-235 suppress worm development from the third-stage larvae to the fourth-stage larvae in *Haemonchus contortus* [5]. The high expression of miR-71 can also facilitate the development of *Echinococcus multilocularis* in vitro [6]. For lncRNA, in the human flatworm *Schistosoma mansoni*, there are 15 differentially expressed praziquantel-resistant lncRNA and three of them are gender-specific [7]. In the free-living nematode *Caenorhabditis elegans*, mutants of 23 lincRNA produced by CRISPR knockout show one or two physiological phenotypes (location, offspring number, defecation, pharyngeal pumping, egg retention, and developmental delay) of varying levels [8]. For circRNA, in the swine nematode *Ascaris suum*, 1982 and 1978 circRNA were separately identified in the body wall and ovary tissue [9]. Also in *C. elegans*, the lack of circRNA, circ-crh-1, exhibited a significantly longer mean lifespan than the wild type [10]. These reports highlight the significant role of ncRNA in helminths.

*Haemonchus contortus* (the barber’s pole worm) is a highly pathogenic hematophagous nematode that infects billions of ruminants (e.g., sheep, goats and cattle), causing destructive diseases and enormous economic losses of tens of billions of dollars per annum globally [11, 12]. Control of this parasite currently relies on anthelmintic drugs, including benzimidazole, imidazothiazoles and macrocyclic lactones, however, the widespread use of these drugs has resulted in serious drug resistance problems in *H. contortus* [13]. Therefore, it is imperative to discover alternative intervention strategies to control haemonchosis. The intestine is a major organ that is responsible for nematode nutrient digestion and absorption, also involved in other processes, including innate immunity, body size control, stress responses and aging [14]. Besides, intestinal proteins have been demonstrated to be capable of inducing high protection against *H. contortus* [15, 16]. Nevertheless, the paucity of molecular and cellular functions in the intestine and the limitations of experimental models are dominating obstacles to developing novel therapeutic targets. Currently, substantial studies have shown that ncRNA perform important biological functions in many pathogenic microorganisms, including bacteria and viruses [17, 18], but the ncRNA remain poorly studied in parasitic nematodes.

Here, we define the ncRNA expression profiles of the *H. contortus* intestine, in order to explore their potential functions and mediated networks. This work provides the most comprehensive accounting of intestinal transcripts (lncRNA, circRNA, miRNA, and mRNA), which is important for facilitating further molecular, biochemical and physiological investigations of *H. contortus* and related nematodes.

## Materials and methods

### *Haemonchus contortus* maintenance and intestine procurement

The three experimental goats (3–6 months) purchased from the Hubei Academy of Agricultural Sciences were confirmed helminth-free by fecal examination and maintained under parasite-free conditions. They were then orally infected with 8000 infective third-stage larvae of Haecon-5 strain of *H. contortus* [19]. On 30 to 35 days after infection, the female adult worms with distinctive appearances [20] were harvested from the abomasum when infected goats were euthanized and necropsied. The collected and live adult females were placed into sterile physiological saline and washed thoroughly. Under the dissection microscope, the intestines intertwined with the reproductive tract were dissected, then the intestines were collected and transferred to liquid nitrogen and frozen for RNA extraction.

### RNA extraction, library construction, and sequencing

Total RNA was prepared with TranZol (Simgen, China) from three independent experimental samples (40 intestines per replicate) following the manufacturer’s instructions. RNA purity and concentration were assessed using NanoDrop 2000 (Thermo Fisher Scientific, USA). The integrity was evaluated on the Agilent 2100 Bioanalyzer system (Agilent, USA).

Approximately 5 µg of total RNA from each sample was treated with Ribo-zero™ rRNA Removal Kit (Illumina, USA) to remove rRNA. The remanent RNA was used as a template for generating a strand-specific library and next sequenced on an Illumina NovaSeq 6000 platform (Novogene Biotechnology Co., Ltd. Beijing, China) with 150 bp pair-end reads. The amount of 3 µg total RNA per intestinal sample was applied to the small RNA library construction. The library was generated using NEBNext® Multiplex Small RNA Library Prep Set for Illumina® (NEB, USA) according to the recommendations, and subsequently sequenced on the Illumina NovaSeq 6000 platform (Novogene Biotechnology Co., Ltd. Beijing, China) and 50 bp single-end reads were generated. The samples were named Gut_1, Gut_2 and Gut_3, respectively. All the raw FASTQ data of RNA-Seq (accession number: PRJNA881596) and small RNA sequencing (Accession number: PRJNA881597) were uploaded to the National Centre for Biotechnology Information (NCBI) database.

### Reads quality control and transcript assembly

The clean data were generated by wiping off the adaptor contamination, reads with poly-N, and low-quality reads from raw data. The software FastQC was used for a quality check on clean data, and during this process, Q20, Q30, and GC content were also calculated. The *H. contortus* reference genome and annotation file were downloaded from Wormbase Parasite database [21] and the filtered data were then aligned using Bowtie2 [22] and/or BWA [23]. The mapped reads were assembled employing StringTie [24] with default parameters.

### Identification and quantification of lncRNA

To identify candidate lncRNA, main filter steps were followed: (1) Transcripts with an exon number of ≥ 2 were selected; (2) Transcripts with a length of < 200 bp were deleted; (3) Transcripts overlapping with the exon region of the annotated database were filtered out; (4) Transcripts with FPKM (Fragments Per Kilobase of exon model per Million mapped fragments) < 0.5 were removed; (5) Transcripts with coding potential were eliminated using CPC [25], CNCI [26] and Pfam-scan [27] software analyses; (6) Transcripts were identified in all three parasite samples. Expression level FPKM for coding transcript and lncRNA was calculated using StringTie [28]. The lncRNA basic features, including classification, exon number, length, open reading frame (ORF) length and expression level, were characterized using R and R Studio.

### Identification and quantification of circRNA

Twenty-nt anchors extracted from both ends of the unmapped reads of Bowtie2 were re-aligned independently with the *H. contortus* reference genome. The anchor sequences that aligned in the reverse direction (head-to-tail) implied circRNA splicing and subsequently submitted to find_circ [29] with default parameters to identify circRNA. The mapped reads of BWA were further detected and annotated using CIRI2 [30] with default parameters. Finally, candidate circRNA were identified using the two algorithms and had at least two back-spliced reads. The expression level of circRNA was calculated and normalized as TPM (transcript per million) [31] based on the following formula: Normalized expression level = (read count ∗ 1 000 000)/libsize (libsize is the sum of circRNA read counts). The classification, genomic localization, exon number, transcript length, and alternative event of predicted circRNA were also characterized using R and R Studio.

### Identification and quantification of miRNA

The clean reads with length between 18 nt to 35 nt were selected for the downstream analyses, and mapped to the *H. contortus* reference genome using Bowtie 2. These mapped reads were blasted against the mature *H. contortus* miRNA sequences from miRbase database (22.1) [32] to identify known miRNA. By searching against the Rfam database [33], the ribosomal RNA (rRNA), transfer RNA (tRNA), small nuclear RNA (snRNA) and small nucleolar RNA (snoRNA) were excluded for further analysis. The integrated software miREvo [34] and mirdeep2 [35] were used to discern the novel miRNA by analysing the secondary structure, the Dicer cleavage site, and the free energy. The expression level of miRNA was normalized by TPM through the following criteria: Normalized expression = mapped readcounts/total reads ∗ 1 000 000. The basic features of miRNA were shown by R and R Studio.

### Functional analysis of target genes of lncRNA and miRNA as well as parental genes of circRNA

Biological functions were assigned to the target genes of lncRNA and miRNA as well as the parental genes of circRNA based on Gene Ontology (GO) analysis and Kyoto Encyclopedia of Genes and Genomes (KEGG) databases. Functional Gene Ontology (GO) analysis was implemented using the g: Profiler [36], a public web server for characterizing and manipulating gene lists. In addition, KEGG pathways were performed by BLAST (E-value < 10 − 5) using *C. elegans* orthologs, which were afterwards assigned to pathways using clusterProfiler [37]. The significantly enriched pathways or GO terms that possessed a padj < 0.05 were plotted with R Studio and the ggplot2 package.

### Construction of ceRNA network

Based on the ceRNA assumption, the circRNA/lncRNA-miRNA-mRNA interaction pairs were predicted by miRanda algorithm [38] with maximum binding-free energy values < − 20 and a match score of 150 or higher. The competing endogenous RNA networks among circRNA/lncRNA, miRNA and mRNA were constructed and visualized using Cytoscape 3.7.2 software.

### Conservation analysis of circRNA and miRNA

The miRNA sequence was severally downloaded from miRbase [32]. The circRNA of adult female worms was previously identified by our group [39]. The sequence conservation of intestinal ncRNA was analyzed through BLASTN (with a threshold of *E* value < 10^−5^).

### Validation of lncRNA, cirRNA and miRNA

The total RNA was isolated from intestinal tissue, and then cDNA was synthesized with HiScript II Q RT SuperMix (Vazyme, China) for circRNA and lncRNA validation. In addition, a 1st Strand cDNA Synthesis Kit (by stem-loop) (Vazyme, China) was used for miRNA verification, following the manufacturer’s protocol.

For putative lncRNA validation, primers were designed for eight randomly selected lncRNA (lnc_004977, lnc_005339, lnc-002272, lnc_001057, lnc_007225, lnc_002287, lnc_002863, and lnc_003171) and afterwards target fragment was amplified by polymerase chain reaction (PCR). PCR was carried out with a 50 µL volume reaction mixture containing 6 µL cDNA, 12 µL ddH_2_O, 2 µL each primer, 1.5 µL dNTP Mix, 1.5 µL Phanta® Max Super-Fidelity DNA Polymerase and 25 µL 2 × Phanta® Max Buffer (Vazyme, China). The PCR profile was as follows: pre-denaturation at 95 ℃ for 3 min, 35 cycles of 15 s at 95 ℃, 15 s at the corresponding annealing temperature and 72 ℃ for 30s, and a final elongation step at 72 ℃ for 5 min. The amplification product was examined by 1% agarose gel and purified with the EasyPure Quick Gel Extraction Kit (TransGen, China). The fragment was subcloned into a pTOPO vector (Aidlab, China), and positive colony was identified and sequenced.

For circRNA verification, divergent primers were designed for eight circRNA to validate the back-splicing junction site using RNase R treated RNA and untreated RNA as templates. The total RNA was incubated with 4 U/µg of RNase R (RNR07250, Epicentre) for 15 min at 37 °C, 10 min at 70 °C, and mock treatment was carried out in the same conditions without RNase R. PCR was performed in a 20 µL volume reaction containing 3 µL cDNA, 5 µL dd H_2_O, 1 µL forward primer, 1 µL reverse primer, and 10 µL 2×Taq Master Mix (Vazyme, China). The PCR program consisted of a pre-denaturation at 94 ℃ for 5 min, followed by 40 cycles including 30 s at 94 ℃, 30 s at the relevant annealing temperature and 72 ℃ for 30 s, and last step by 10 min at 72 ℃. The PCR product was checked on 1% agarose gel and sent to the company for Sanger sequencing.

To verify miRNA, stem-loop RT primers and forward primers were designed for 12 miRNA (five known and seven novel). The cDNA for each miRNA was synthesized employing a corresponding stem-loop primer. PCR system and procedure were similar to those of circRNA validation and the product was examined with 1% agarose gel and captured image. All the synthesis of primers (Additional file 1) and sequencing for validation of lncRNA, cirRNA, and miRNA were completed by TSING Ke Biotech Co., Ltd. (Wuhan, China).

## Results

### Isolation of the intestines of adult worms

The intestines were dissected from adult female worms under the dissection microscope. Two images of the separated intestine are shown in Additional file 2. Additional file 2A is an optical micrograph of female *H. contortus*, with the posterior section of the vulval flap removed to allow the intestine to spill from the carcass. Additional file 2B shows the intestine separated from the reproductive tract.

### Identification and genomic features of intestinal lncRNA

Three strand-specific libraries were constructed by removing rRNA from the total RNA of the three intestinal samples (Gut_1, Gut_2 and Gut_3) and sequenced, respectively. Overall, 17.09G, 14.97G and 16.96G clean bases were obtained, corresponding to 113.9, 99.8, 113.1 million clean reads. G20, G30 and GC content are also presented. For the three libraries, 64.24%, 66.8% and 62.37% of gained reads could be successfully mapped to the *H. contortus* reference genome (Additional file 3).

**Figure 1:**
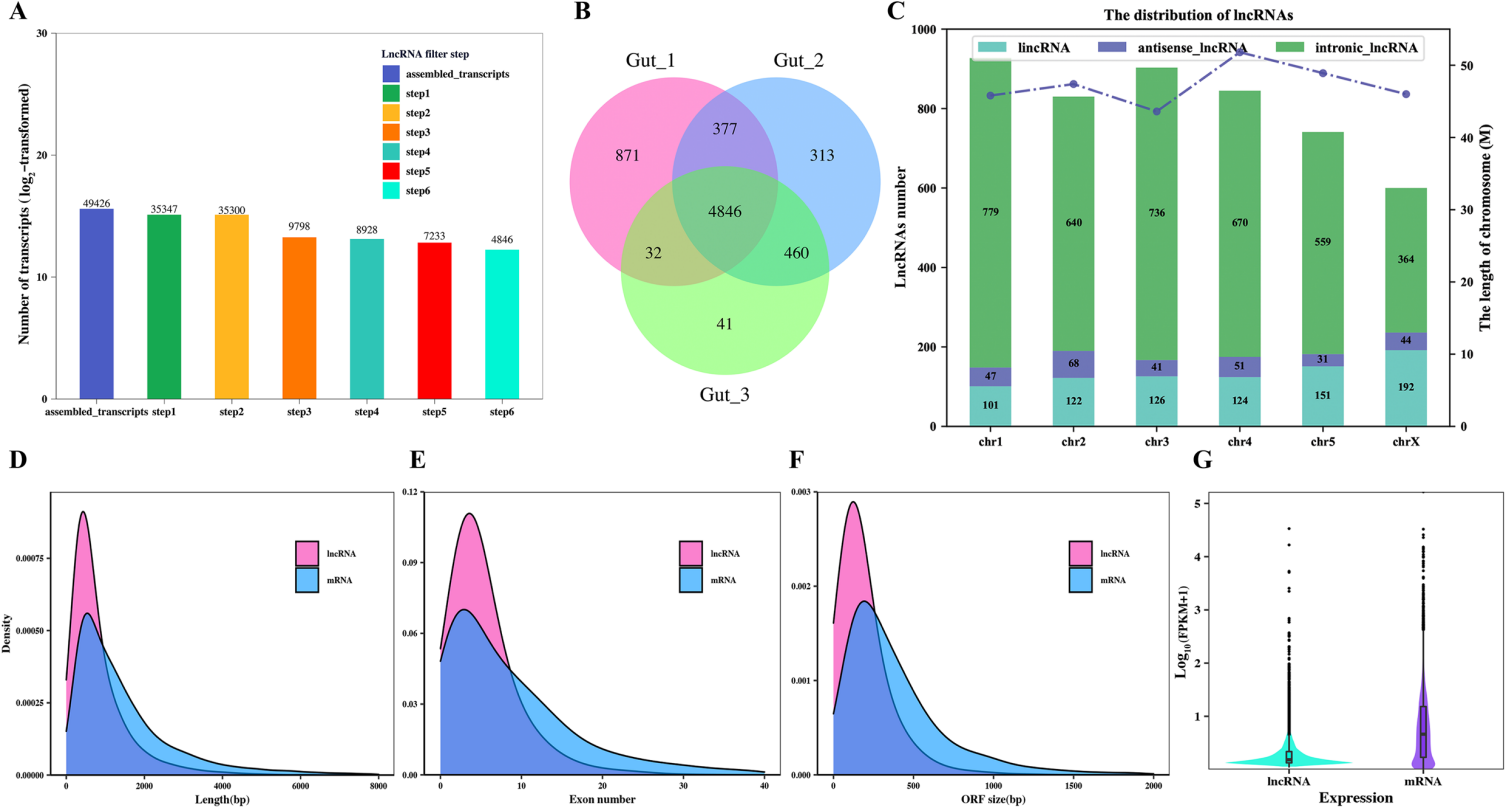
**Identification of female intestinal** ***Haemonchus contortus *lncRNA and comparison of characteristics of lncRNA and mRNA.** **A** Identification of lncRNA in *Haemonchus contortus* female intestine. **B** The Venn diagram of the potential lncRNA transcripts screened from the three intestinal samples. **C** The histogram showing the number of lncRNA detected in six *H. contortus* chromosomes. (**D**-**F**) The density plot reveals the comparison of length (**D**), exon numbers (**E**), and open reading frame (ORF) length (**F**) between lncRNA and mRNA. **G** The violin plot shows the expression levels of lncRNA and mRNA.

According to the lncRNA filtering steps, a total of 4846 reliable novel lncRNA were predicted (Figures 1A and B, Additional file 4). In addition, 8821 mRNA were also obtained in the study (Additional file 4). Based on lncRNA distribution in the genomic regions, the identified lncRNA were classified into three types, including 3748 (77.34%) intronic_lncRNA, 282 (5.82%) antisense_lncRNA, and 816 (16.84%) lincRNA (Figure 1C). We observed that lncRNA were widely distributed on each *H. contortus* chromosome, and a majority of them localized on chromosome 1, while the distribution on chromosome X was the least (Figure 1C). The lengths of lncRNA were shorter than those of mRNA, with the former ranging from 202 bp to 13 892 bp with the median length of 738 bp, and the latter ranging from 160 bp to 28 635 bp with the median length of 1545 bp (Figure 1D). A comparison of the exon numbers between lncRNA and mRNA suggests lncRNA had a higher density than mRNA when exons were under eight, so lncRNA possess fewer exon numbers than mRNA in the *H. contortus* genome (Figure 1E). In terms of the size of predicted ORF, those encoded by lncRNA were also shorter than those encoded by mRNA (Figure 1F). Furthermore, the analysis of the global expression level of lncRNA shows that it was lower than that of mRNA (Figure 1G).

### Identification and genomic features of intestinal circRNA

**Figure 2:**
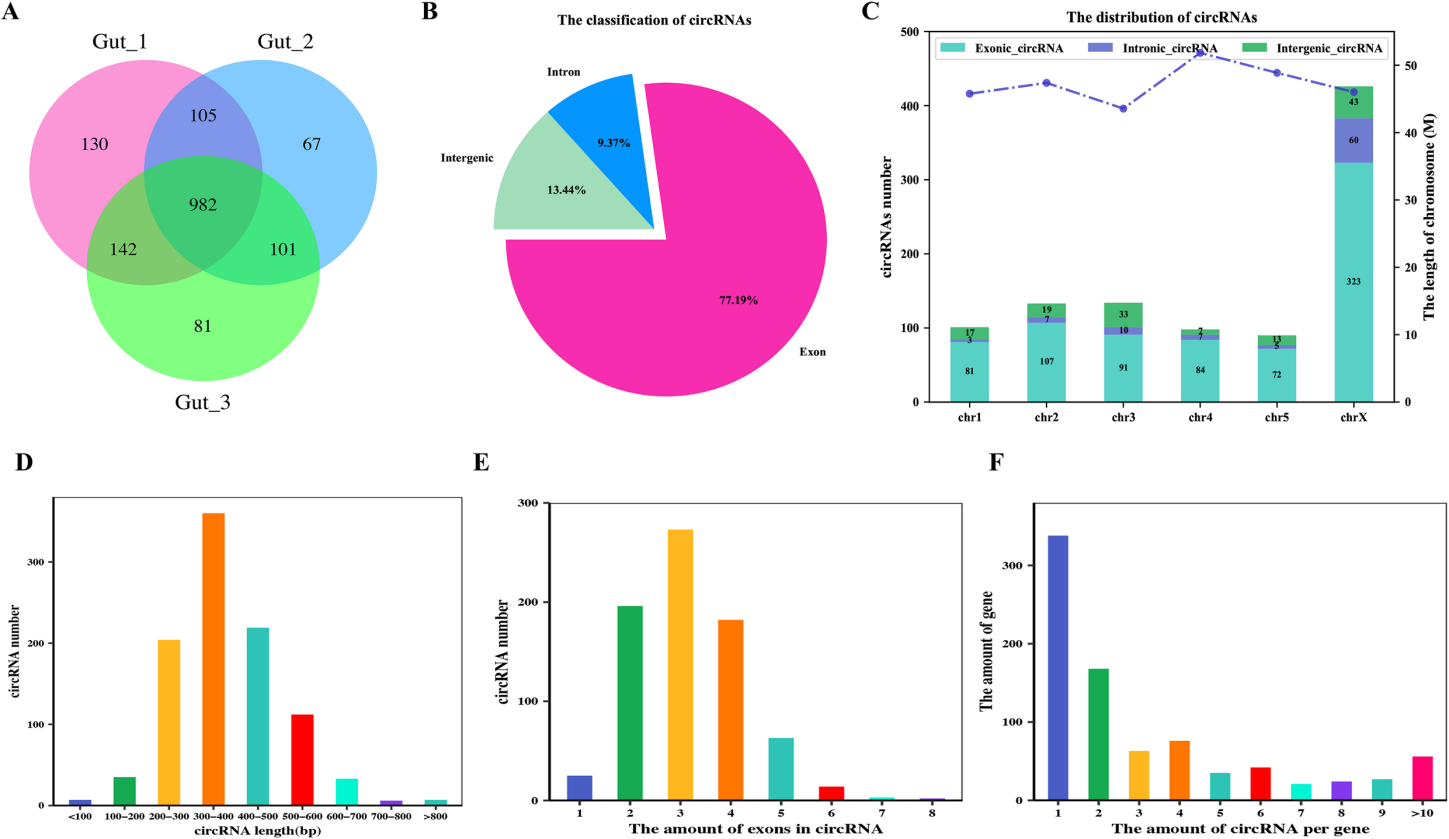
**The features of circRNA identified from the intestine of female** ***Haemonchus contortus***. **A** The Venn diagram of the potential circRNA transcripts screened from the three female worm intestinal samples. **B** The pie chart represents the amount and percentage of circular RNA generated from exonic, intronic, and intergenic regions. **C** The histogram represents the distribution of circRNA in six *H. contortus* chromosomes. **D**–**E** The bar plot shows the length (**D**), the amount of exon (**E**), and alternative event (**F**) of identified intestinal circRNA, respectively.

The identification of circRNA was carried out based on the constructed lncRNA library. A total of 982 candidate circRNA with more than two back-spliced junction reads were identified and annotated using find_circ and CIRI2 computational pipelines (Figure 2A, Additional file 4). There were also three major categories of the putative circRNA, namely 758 (77.19%) exonic circRNA, 92 (9.37%) intronic region, and 132 (13.44%) intergenic circRNA (Figure 2B). The results show that these circRNA were widely and unevenly distributed across the whole *H. contortus* chromosomes, and were independent of chromosome length. On the contrary to lncRNA, circRNA were more abundantly distributed on chromosome X than on other chromosomes (Figure 2C). The average length of circRNA was 356 bp, while the maximum length was 1085 bp. Most of the circRNA (91.14%) ranged from 200 to 600 bp lengths (Figure 2D). The analysis revealed that the vast majority of exon-derived circRNA encompass two to four exons (Figure 2E). Moreover, these 982 circRNA were derived from 489 parental genes, of which 69.12% produced only one circRNA, and the remainder produced more than one circRNA (Figure 2F) with HCON_00177780 (sma-9) producing the largest number of circRNA (*n* = 18).

### Identification and characteristics of intestinal miRNA

**Figure 3:**
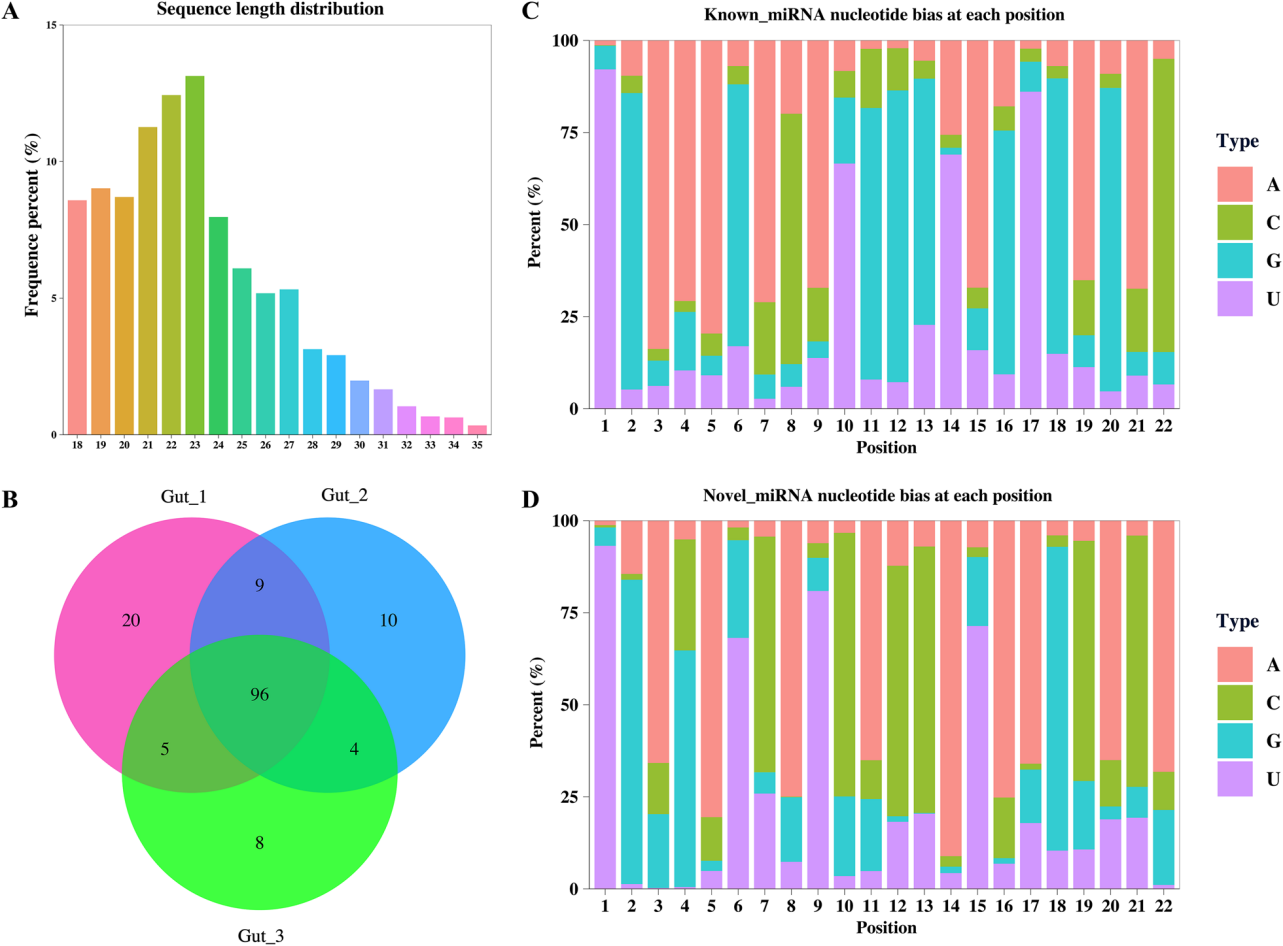
**The features of miRNA identified from the intestine of female *Haemonchus contortus***. **A** Length distribution of small RNA of female worm intestine. **B** The Venn diagram of the potential miRNA screened from the three female worm intestinal samples. **C** The stack column plot shows nucleotide bias at each nucleotide position of known miRNA. **D** The stack column plot represents nucleotide bias at each nucleotide position of novel miRNAs.

For the small RNA libraries, 17.7, 25.3, and 16.8 million raw reads were identified, and the corresponding 16.7, 24.8, and 16.6 million clean reads were obtained after filtering the low-quality reads, respectively. Afterwards, 7.8, 17.4, and 12.2 million reads were generated with trimmed lengths between 18 and 35 nt. The length distribution revealed that most miRNA were 21–23 nt long and the predominant length was 23 nt (Figure 3A). 90.54%, 95.56% and 95.86% of reads were mapped to the genome with Bowtie2 (Additional file 3). After rRNA, tRNA, snRNA, and snoRNA were filtered out, a total of 96 mature miRNA were obtained including 65 known and 31 novel miRNA (Figure 3B, Additional file 4). Among both the known and novel miRNA, the preferred first nucleotide base was U, with 92.1% for known miRNA (Figure 3C), and 93.1% for novel ones (Figure 3D). Among identified known miRNA, the 15 most abundant miRNA included miR-71, miR-5885a, miR-9, miR-5885b, miR-5976, miR-5960, miR-5908-3p, miR-993, miR-259, miR-60, miR-50, lin-4, miR-5895, miR-83, and miR-61 (Additional file 5). For novel miRNA, the length of the pre-miRNA (precursor miRNA) occurred at a range of 44 to 78 bp and the average length was 58 bp (Additional file 4). All the predicted novel miRNA were confirmed with their characteristic stem-loop structures. Nine novel intestinal miRNA (novel-miR-2/5/8/16/22/33/60/69/75) shared seed sequences (bases 2–8) with known miRNA of *H. contortus*. Six novel miRNA (novel-miR-19/20/45/53/72/89) shared seed sequences with miRNA of other nematodes (*Ascaris suum*, *Brugia malayi*, *Caenorhabditis elegans*, *Heligmosomoides polygyrus*, *Strongyloides ratti*) in the miRBase. In addition, four novel seeds (novel-miR-101/25/29/49) were identified (i.e., no miRNA in miRBase v22.1 has any of these seed sequences). Among novel miRNA, the highly expressed nine miRNA in the intestine were displayed in Additional file 5. Comparing miRNA derived from the intestine and EV (40 miRNAs) [43], there were 28 miRNA existing in both data (Additional file 6).

### Validation of circRNA, lncRNA and miRNA

**Figure 4:**
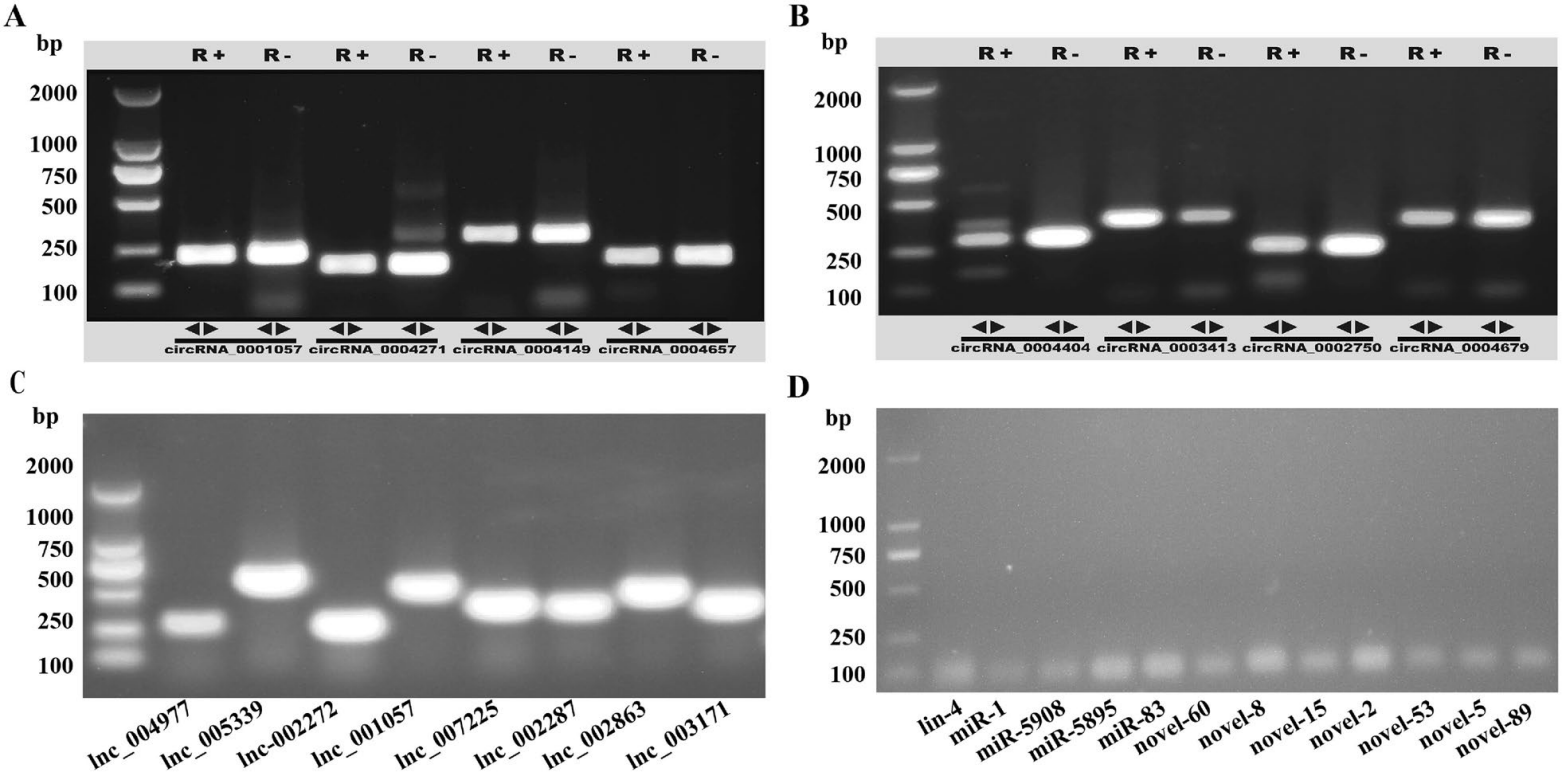
**PCR validation of female** ***Haemonchus contortus*** **intestinal circRNA, lncRNA, and miRNA**. **A** and **B** The agarose gel electrophoresis shows PCR products amplified with primers designed based on the DNA sequence across the back junction site of each indicated circRNA. R+: with RNase R digestion, R-: without RNase R digestion, DL2000 Marker; **C** The agarose gel electrophoresis of PCR amplified eight lncRNA. DL2000 Marker, lnc_004977, lnc_005339, lnc-002272, lnc_001057, lnc_007225, lnc_002287, lnc_002863, and lnc_003171 from left to right, respectively. **D** The agarose gel electrophoresis of PCR amplified 12 intestinal miRNAs. DL2000 Marker, lin-4, miR-1, miR-5908, miR-5895, miR-83, novel-60, novel-8, novel-15, novel-2, novel-53, novel-5, novel-89 from left to right, respectively.

Eight circRNA, lncRNA and twelve miRNA were randomly selected to validate the data of deep sequencing. All eight selected circRNA were amplified with the desired size of the target fragment (Figures 4A and B) and the junction sites were verified by Sanger sequencing (Additional file 7A). On the contrary, the product size of RNase R-treated RNA was similar to that of mock-treated RNA, indicating the resistance of circRNA to RNase R digestion. In addition, LncRNA fragments were subcloned into the pTOPO vector for sequencing analysis (Figure 4C). Lnc_005339, lnc_007225 and lnc_002287 sequences were consistent with high-throughput sequencing results, and lnc_001075, lnc_002272, lnc_003171 and lnc_004977 had less than 5 base mutations (Additional file 7B). Although 14 bp sequence differences occurred in lnc_002863, the reason may be due to the individual differences of the strain. Lastly, agarose gel electrophoresis revealed that the product size of all selected miRNA (5 known and 7 novel) was fully matched (Figure 4D). The experimental results were in good accordance with high-throughput sequencing. Overall, the sequencing assembly and identification process is reliable.

### Functional analysis of lncRNA, miRNA target genes and circRNA parental genes

**Figure 5:**
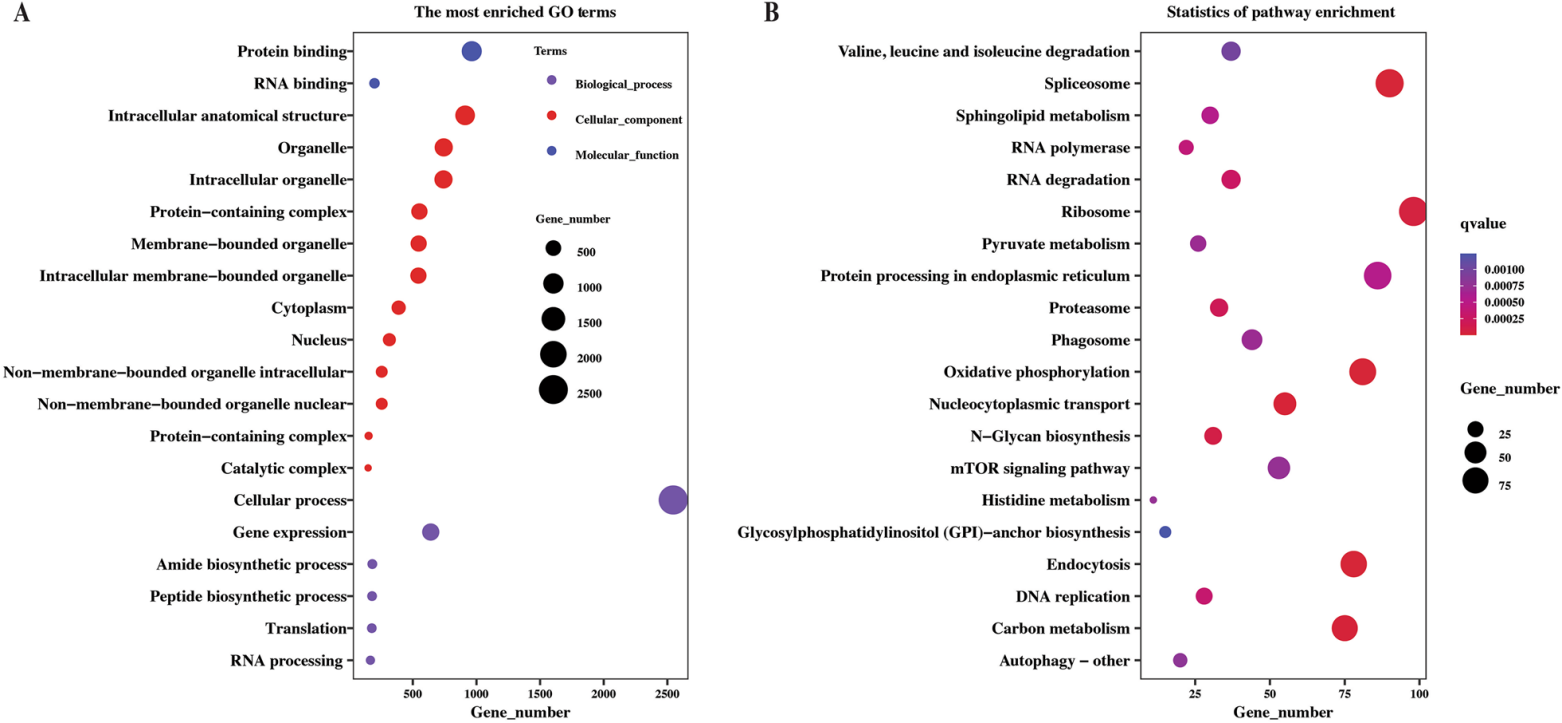
**Functional analysis of the** ***cis*-lncRNA target genes of lncRNA identified from the intestine of female *Haemonchus contortus***. Top 20 significantly enriched GO terms (**A**) and KEGG pathways (**B**) for target genes of lncRNA. Each scatter point represents a GO term and pathway. The size of each point represents the enriched gene number. The color of each point represents the GO categories and the size of the q value.

To explore the role of lncRNA, the target genes were predicted based on the hypothesis that lncRNA could regulate their neighboring target genes in *cis*. The downstream and upstream 100 kb coding genes of lncRNA were searched to analyze their functions. A total of 8265 target genes could be cis-regulated by 4826 lncRNA (Additional file 8). The functional association of *cis*-lncRNA target genes identified 154 different GO terms (33 for molecular functions, 81 for biological processes, and 40 for cellular components) (Additional file 9). They were involved in intracellular anatomical structure, organelle, and intracellular organelle of cellular components. In the molecular function category, most genes mainly exerted protein and RNA binding activity. While in the biological process, the cellular process and gene expression were enriched to the greatest number of genes (Figure 5A). Meanwhile, the KEGG pathway analysis indicated that *cis* target genes were enriched in 67 pathways, mainly including spliceosome, carbon metabolism, proteasome, endocytosis, and biosynthesis of amino acid pathway (Figure 5B, Additional file 9).

**Figure 6:**
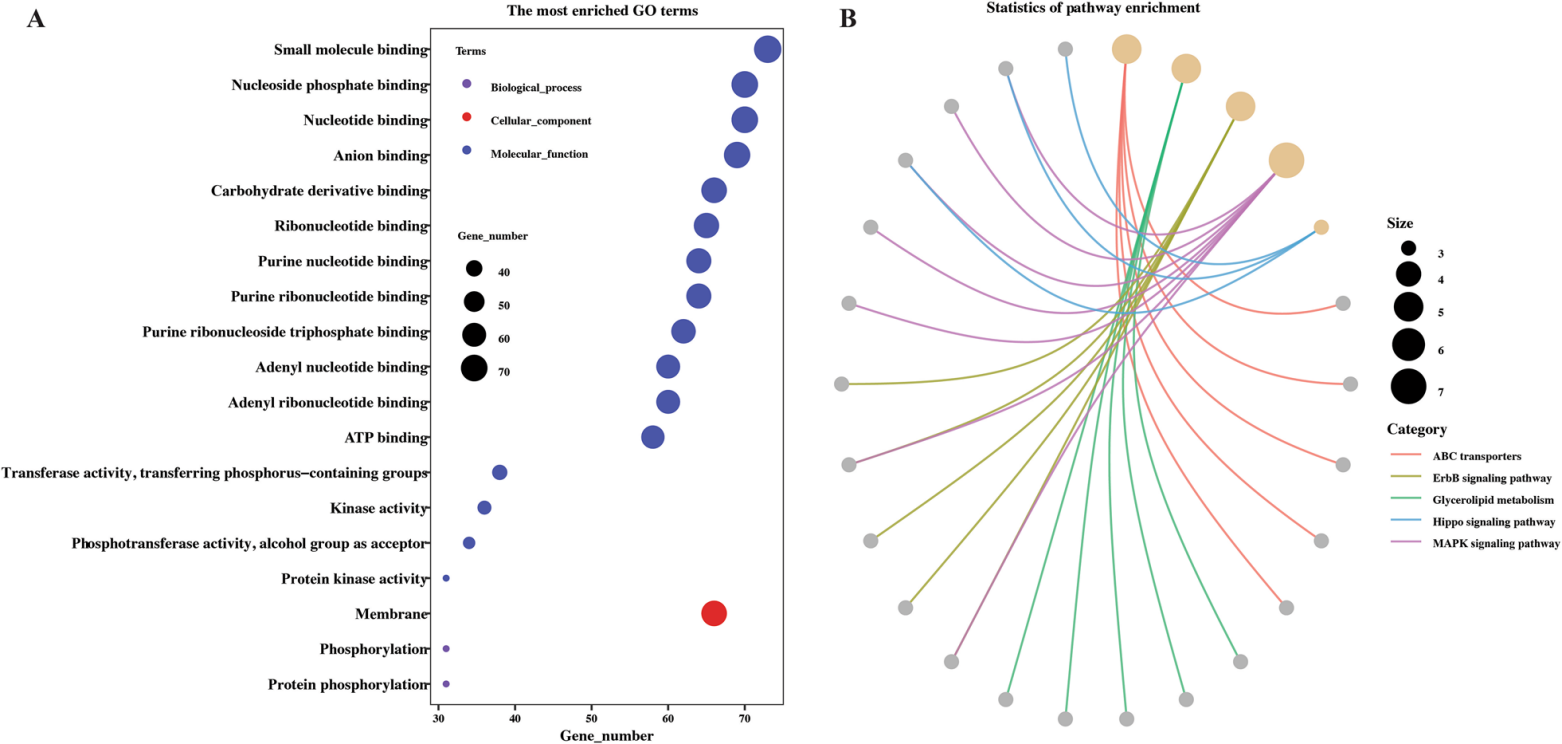
**Functional analysis of the parental genes of circRNA identified from the intestine of female *Haemonchus contortus***. **A** The significantly enriched GO terms. Each scatter point represents a GO term and the size of the point represents the gene number. The color of each point represents the GO categories. **B** The significantly enriched KEGG pathways. The size of the point and the color of the line represent the gene number and pathway, respectively.

The GO annotation indicated that the circRNA parental genes were primarily related to the binding activity, such as small molecular binding, ribonucleotide binding, nucleotide binding, and ATP binding. While only membrane, protein phosphorylation and phosphorylation enriched in cellular components and biological processes, respectively (Figure 6A, Additional file 9). The KEGG pathway analysis shows that the source genes were significantly enriched in ABC transporters, ErbB signaling pathway, glycerolipid metabolism, hippo signaling pathway and MAPK signaling pathway (Figure 6B, Additional file 9).

**Figure 7:**
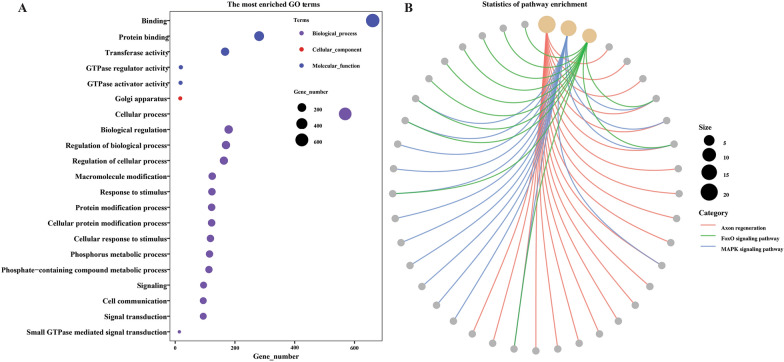
**Functional analysis of the target genes of miRNA identified from the intestine of female *Haemonchus contortus***. **A** The significantly enriched GO terms. Each scatter point represents a GO term and the size of point represents the gene number. The color of each point represents the GO categories. **B** The significantly enriched KEGG pathway. The size of the point and the color of the line represent the gene number and pathway, respectively.

To predict mRNA targets for miRNA, 6795 genes were estimated that 3’ UTR sequences (> 100 bp) were sufficient for miRNA target prediction. GO analysis shows that the majority of targeted reference mRNA biological process mainly included cellular process, cellular response to stimulus, cellular protein modification process, and signal transduction, while the molecular function of targeted mRNA was involved in binding, protein binding, and transferase activity. Among cellular components, mRNA targets were only enriched in Golgi apparatus (Figure 7A, Additional file 9). The KEGG pathway enrichment denoted that the targeted mRNA genes were mostly associated with Axon regeneration, Foxo signaling pathway, and MAPK signaling pathway (Figure 7B, Additional file 9).

### CeRNA network construction

LncRNA and circRNA act as competitive endogenous RNA (ceRNA) and are mutually regulated by competition for binding to common microRNA response elements (MRE). The possible interaction network of lncRNA and circRNA with miRNA was predicted and constructed through a miRanda algorithm (Additional file 10). A total of 5304 relationship pairs were predicted between 2546 lncRNA and 96 miRNA, among which LNC_007002 harbored the most binding sites (23) with novel-miR-45. The 356 relationship pairs with 1 or 2 binding sites between specific pairs were generated, including 277 circRNA and 82 miRNA. Meanwhile, a total of 1678 mRNA transcripts were predicted to be targeted by 94 intestinal miRNA, and 2739 relationship pairs were obtained. However, most relationship pairs have only 1 or 2 binding sites between them, and just 39 miRNA-mRNA relationship pairs contain 3 or 4 binding sites.

## Discussion

*H. contortus*, is a blood-feeding parasite that causes substantial mortality and morbidity in livestock animals, resulting in major losses in agricultural production [11]. With the problem of drug resistance increasing, there is an urgent need to develop novel prevention and control strategies against this parasitic nematode. *H. contortus* intestine is responsible for nutrient digestion and absorption and has the powerful potential to be a valuable target for anthelmintic drug and/or vaccine development [40, 41]. Previous publications have explored the intestinal cell biology through transcriptome [14, 42] and proteomics [15] of *H. contortus*, but the ncRNA remain poorly studied. Hence, the present study was to provide the full-scale compilation of intestinal transcripts (lncRNA, circRNA, miRNA and mRNA) and their corresponding functional categories.

A total of 4846 lncRNA and 8821 mRNA were identified in the intestine of *H. contortus* and genomic features of lncRNA were characterized by comparing with mRNA. The result revealed that the sequence length, ORF length, chromosome distribution and exon number of lncRNA of *H. contortus* resembled those of multiple organisms, such as mammals [43], plants [44], arthropods [45], and parasites [46], indicating the consistency of basic characteristics of lncRNA among different species. In addition, the expression levels of lncRNA were lower than those of mRNA, which is also consistent with the results from other studies [47]. Nevertheless, several studies have shown that although the expression levels of lncRNA are low, they still play important roles in many biological processes [47]. The basic characteristics of intestinal circRNA, including classification, distribution on the chromosome, exon number, and spliced length, follow those of circRNA identified from three stages/sexes (infective third-stage larvae, female, and male adult worm) of *H. contortus* in our previous study [39].

Although the expression profile of intestinal miRNA of *H. contortus* was previously analyzed by microarray [5], the construction of an intestinal tissue-specific small RNA library could observably contribute to mining a more complete miRNA expression profile. Here, among the 31 identified novel miRNA, novel-miR-2 belonging to the hco-miR-5885 family with the highest expression level was homologous to the highly expressed miR-58 of *C. elegans*. In *C. elegans*, the miR-58 family not only regulated longevity through the insulin signaling pathway [48], but also played a vital role in growth and stress resistance via the interactions with TGF-β genes [49]. In *S. japonicum*, inhibiting of miR-bantam (one member of the miR-58 family) could lead to ovary morphological changes, suggesting that it has a crucial function in the sexual maturation and oviposition [50]. Meanwhile, this miRNA is a potential biomarker for schistosome infection because it can be detected in the host serum [51]. In addition to the regulatory roles in the parasite’s biology, most importantly, the miRNA could regulate the expression of host genes and help the parasite survive in the host [52]. The sequence of novel-miR-53 with the second highest expression level among 31 identified novel miRNA was homologous to miR-34 of *C. elegans*. In *C. elegans*, the miR-34 family is a multifunctional miRNA involved in tissue development, aging, stress response, spermatogenesis and signal transduction [53]. In the present study, the novel-miR-53 of *H. contortus* with the second highest expression level among 31 identified novel miRNA was identical to miR-34 of *C. elegans*. In addition, four novel seed sequences (novel-101/25/29/49) of *H. contortus* were identified. Besides these novel miRNA, known miR-60, an intestinal miRNA in *C. elegans*, modulates the adaptative response to chronic oxidative stress [54]. In *H. contortus*, miR-60 is also highly expressed in intestine and parasitic-living larvae [5], therefore, this miRNA likely contributes to intestinal development and adapts to the transformation of the environment more quickly. Although little is known about the functions of miRNA in *H. contortus*, the conservation between miRNA of *H. contortus* and those of *C. elegans* suggest that miRNA were likely involved in various biological and physiological processes, but the specific roles of miRNA still need further functional studies.

Over the past years, there has been an explosion in the knowledge of extracellular vesicles (EV) that modulate the host immune response [55]. Employing multiple omics technology, it revealed that EV cargo from both eukaryotic and prokaryotic cells was enriched with miRNA, lncRNA, circRNA, proteins, and other putative effectors [56, 57]. Corresponding advances have been made in helminth extracellular vesicles and most of the focus has been on the miRNA and proteins in the helminth-host interaction to promote parasite survival. In the mouse parasitic nematode *H. polygyrus*, there were abundant seed sequences of miRNA within EV which induced protective immunity and were identical to those of *M. musculus* miRNA, including *lin-4*, *let-7*, miR-63, miR-79, miR-100, etc. [58]. In *A. suum*, the helminth-infection hallmark cytokines IL-13, IL-25 and IL-33 were identified as potential targets of *lin-4-5p* and *let-7-5p*, miR-5350d-5p and miR-87a/b-3p, respectively [59]. In *S. japonicum*, miR-125 and *bantam*, miRNA from EV can regulate host macrophages to facilitate parasite survival [52]. Interestingly, the miRNA released from EV of *Ascaris suum* [60] and *H. contortus* [61] were also determined in the corresponding gut tissue, suggesting the potential intestinal origin of some nematode miRNA found in EV. Here, the hypothesis was reinforced because comparing miRNA derived from the intestine and EV (40 miRNA), there were 28 common miRNA. Of course, some miRNA are found in EV with lower expression in the gut tissue, such as the hco-miR-5352 cluster (hco-miR-61, hco-miR-5352, hco-miR-43 and hco-miR-5895), indicating they can also possibly be from other tissues [61]. It is intriguing to speculate that these miRNA in *H. contortus* can be delivered to host immune cells to regulate pathogen-host interaction, thereby being important for the initial establishment and survival in the host.

To date, a series of functions and mechanisms have been reported for many mammalian and plant lncRNA [2]. These lncRNA could *cis* regulate the transcriptional expression of upstream and downstream adjacent genes smaller than 100 Kb [62, 63]. In *C. elegans*, the knockdown of some lincRNA, such as lincRNA-17 and lincRNA-18, significantly increased the expression levels of adjacent genes [8], suggesting that this mechanism is also likely to be widespread in parasitic nematodes. Here, 4826 lncRNA were found to *cis* regulate 8265 target genes based on the predicted results, and 154 different GO terms and 67 pathways were enriched, indicating that target genes of lncRNA possibly involved in many biological processes through binding DNA, RNA and protein. Interestingly, 31 target genes were enriched to be associated with N-glycan biosynthesis. Studies have shown that N-glycosylation can greatly contribute to the immunogenicity of proteins of *H. contortus* [64]. In addition, lncRNA can serve as a potential precursor molecule of small RNA [65], and studies have shown that small RNA can be modified by N-glycan [66]. LncRNA can also affect the mucin-type O-glycan biosynthesis, the deficiency of mucin-type O-glycan biosynthesis leads to disruption of intestine homeostasis and to the re-shaping of the entire intestinal microbial community [67]. In addition, KEGG pathway analysis shows that the source genes of circRNA were significantly enriched in the ABC transporters pathway (HCON_00085890, HCON_00098130, HCON_00130050, HCON_00164880, HCON_00189480). Many studies have shown that nematode ATP-binding cassette (ABC) transport proteins play an important role in anthelmintic resistance [68], suggesting that circRNA may have an important role in drug resistance in *H. contortus*.

A growing body of evidence has revealed that lncRNA, circRNA or pseudogenes can act as ceRNA to competitively bind miRNA through miRNA response elements, thereby weakening the inhibition of miRNA on mRNA [69, 70]. Understanding the ceRNA could provide important insights into the regulation of gene expression. The predicted results show that 94 miRNA targeted 1678 mRNA transcripts, 82 miRNA could competitively bind 277 circRNA, and 2546 lncRNA targeted 96 miRNA. The results of the RNA-mediated interaction network support the speculation that lncRNA and circRNA have great potential as miRNA sponges and may be an intriguing regulatory substance in intestinal cell development of *H. contortus*. Therefore, computational target predictions warrant further experimental validation to provide more conclusive answers.

In summary, this study identified the first comprehensive expression profiles of lncRNA, circRNA, miRNA, as well as mRNA for *H. contortus* intestine. Functional analysis shows that these RNA could be involved in worm survival and/or development. Accurate interaction pairings of lncRNA/circRNA-miRNA-mRNA relationships were constructed from tissue-level resolution. Lastly, comparative analysis between the intestinal miRNA and EV-miRNA supported the perspective that some nematodes EV-miRNA derive from the intestine tract. Our work, therefore, provides the most comprehensive atlas of non-coding and coding transcripts in the intestine of *H. contortus* and lays the foundation for further functional research.

### Supplementary Information

**Additional file 1. Primers used in PCR amplification for validating the selected intestinal lncRNA, circRNA, and miRNA of female *Haemonchus contortus***.

** Additional file** **2. Intestine dissected from female *Haemonchus contortus*** **adult worm.** (A) Female *H. contortus* adult worm optical micrograph, the vulval flap posterior section was cut off to make intestine leave the carcass. (B) Separated intestine and reproductive tract. I: intestine; R: reproductive tract. Scale bar: 20 μm.

** Additional file 3. The output data quality of female *Haemonchus contortus* intestinal lncRNA or miRNA libraries.**

** Additional file 4. The detailed information of identified intestinal lncRNA, circRNA, miRNA, and mRNA of female *Haemonchus contortus.***

** Additional file 5. Highly expressed known or novel intestinal miRNA of female** ***Haemonchus contortus.***

** Additional file 6. The miRNA shared between female worm intestine and EV of *Haemonchus contortus***.

** Additional file 7. The Sanger sequencing result of intestinal circRNA and lncRNA of female** ***Haemonchus contortus***. (A) The Sanger sequencing result of back junction site of eight intestinal circRNA. The vertical line represented junction site. (B) The results of Sanger sequencing of eight selected intestinal lncRNAs.

** Additional file 8. The corresponding information of ***cis***-regulated target genes of intestine of female *Haemonchus contortus* lncRNA.**

** Additional file 9. Functional analysis of the female *Haemonchus contortus* intestinal lncRNA or miRNA target genes and circRNA source genes.**

** Additional file 10. The target site information about intestinal lncRNA/circRNA-miRNA-mRNA of female *Haemonchus contortus.***

## Data Availability

RNA-seq data used and/or analyzed during the current study are available from the corresponding authors upon reasonable request. The datasets supporting the conclusions of this article are included within the article and additional files.
