# Supplementary figures and images for: Non-coding RNA in the gut of the blood-feeding parasitic worm, Haemonchus contortus
Source: Vet Res. 2024 Jan 3;55:1. doi: 10.1186/s13567-023-01254-x (PMC10763314; doi:10.1186/s13567-023-01254-x)

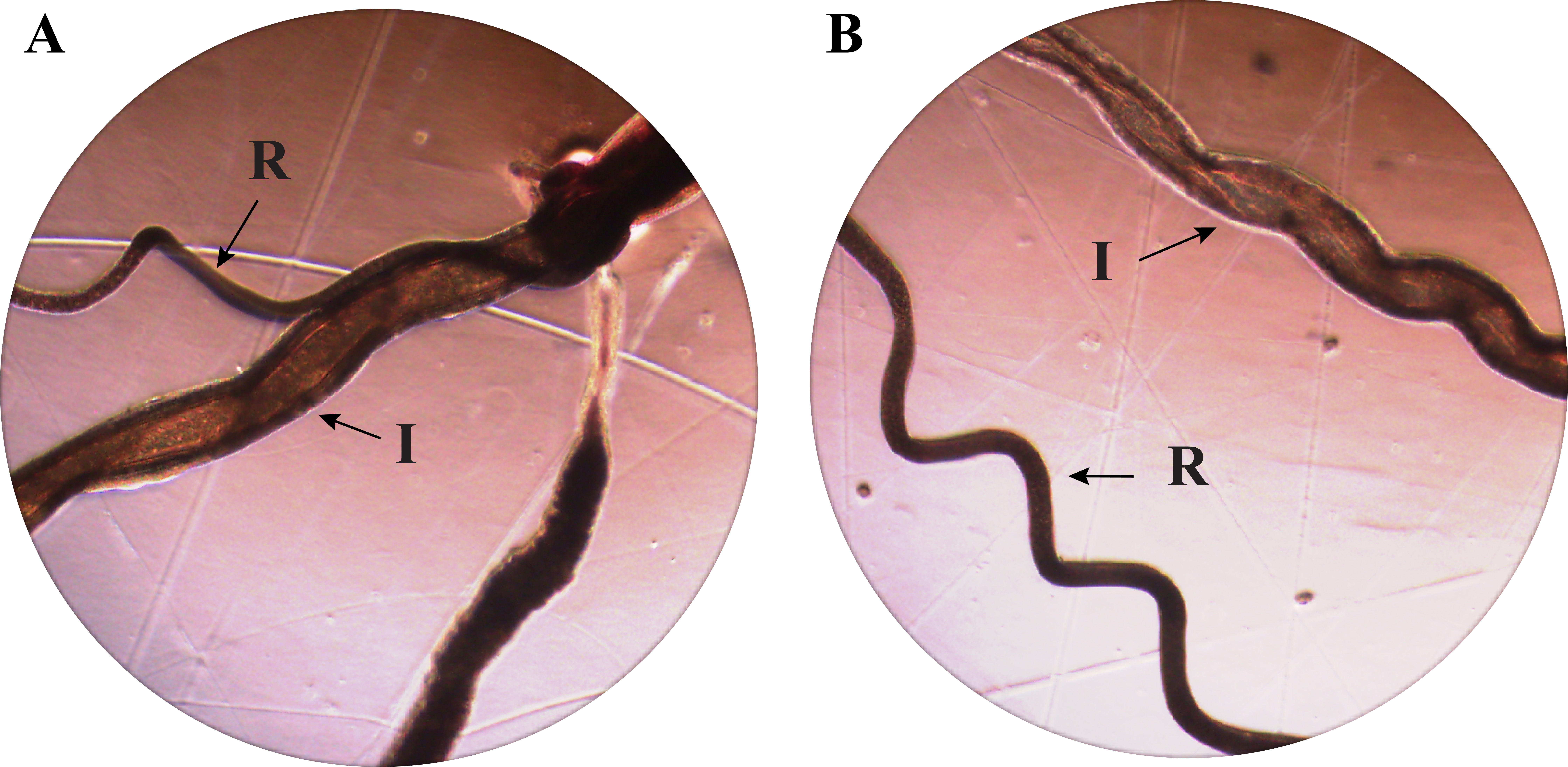

Supplement: Supplementary file 2 — Additional file 2. Intestine dissected from female Haemonchus contortus adult worm. (A) Female H. contortus adult worm optical micrograph, the vulval flap posterior section was cut off to make intestine leave the carcass. (B) Separated intestine and reproductive tract. I: intestine; R: reproductive tract. Scale bar: 20 μm. [file 13567_2023_1254_MOESM2_ESM.jpg]

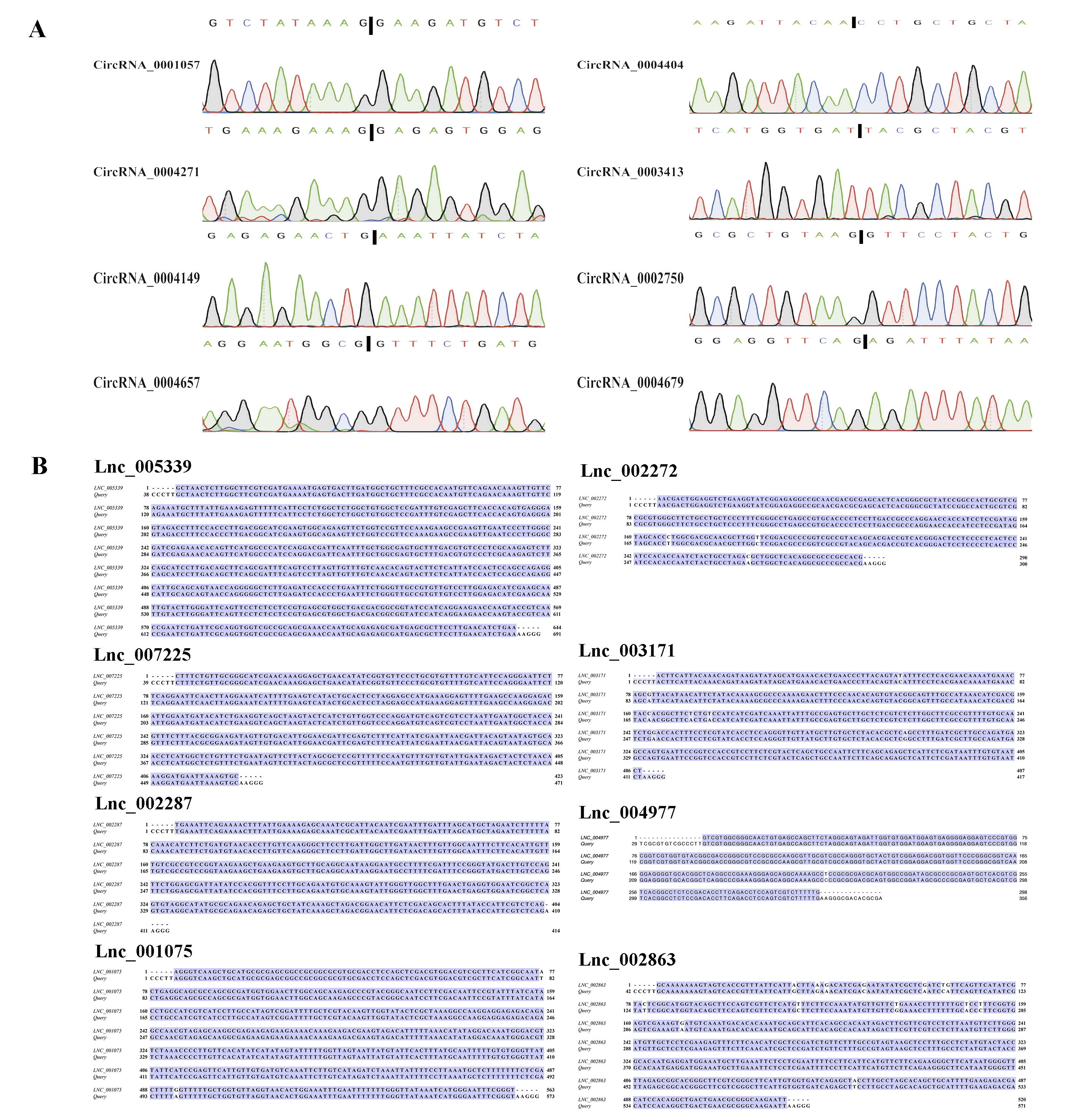

Supplement: Supplementary file 7 — Additional file 7. The Sanger sequencing result of intestinal circRNA and lncRNA of female Haemonchus contortus. (A) The Sanger sequencing result of back junction site of eight intestinal circRNA. The vertical line represented junction site. (B) The results of Sanger sequencing of eight selected intestinal lncRNAs. [file 13567_2023_1254_MOESM7_ESM.jpg]
